# Atopy and related clinical symptoms among Swiss medical students from 2007 to 2015

**DOI:** 10.1186/s13223-018-0230-4

**Published:** 2018-02-02

**Authors:** Lukas Steinegger, Stephan Regenass, Lucas M. Bachmann, Elsbeth Probst, Urs C. Steiner

**Affiliations:** 1 Division of Clinical Immunology, University Hospital Zurich, Gloriastrasse 23, 8091 Zurich, Switzerland; 2 Division of Pathology, Laboratory, University Hospital Basel, Basel, Switzerland; 3 Medignition Inc. Research Consultants, Zurich, Switzerland

**Keywords:** Atopy, Time trends of sensitization, Polysensitization

## Abstract

**Background:**

Atopic allergy is a widespread disease with increasing prevalence in the second half of the twentieth century and is most often associated with clinical symptoms, like rhinoconjunctivitis, asthma or eczema. This study explored the prevalence of atopy and polysensitization in nine cohorts of Swiss medical students during the period of 2007–2015. Furthermore, the self-reported allergic symptoms, such as rhinoconjunctivitis, asthma and eczema, among students with and without atopy were assessed.

**Methods:**

Each cohort was assessed in the third study year. Students underwent an ImmunoCAP rapid test, a qualitative point-of-care test, and completed an anonymous questionnaire on age, gender and clinical symptoms including rhinoconjunctivitis, asthma and eczema. Statistical analyses assessed the overall prevalence of atopy in each group and estimated the average annual increase using a linear mixed model. We examined the frequency of occurrence of polysensitization and differences of reported symptoms among students with and without atopy.

**Results:**

Data of 1513 students (mean age 22.4–23.3 years across cohorts) in nine cohorts (median cohort size 215 interquartile range IQR 193–222) were available for analysis. Test results consistent with atopy were present in 39.9% of students. Average increase of atopy over the 9 years of observation was 2.25% (95% CI 0.18–4.31%; p = 0.037). Main drivers for this increase were the ubiquitously available allergens, house dust mite, timothy grass and birch pollen. Atopy and polysensitization were more pronounced in male students: Polysensitization also increased in the observation period. The clinical symptoms, rhinoconjunctivitis, asthma and eczema were reported by 463 (76.7%) atopic and by 141 (15.5%) non-atopic students.

**Conclusions:**

We observed a slight increase of atopy and polysensitization within 9 years of observation in Swiss medical students. The most frequent sensitization occurred with allergens with the highest chance of exposure. Rhinoconjunctivitis, asthma and eczema are a symptom complex associated with atopy but also found in non-atopic students.

*Trial registration* retrospectively registered by the Cantonal Ethics Committee Zurich on 22.01.2016; Nr: 08-2016

## Background

Atopic allergies belong to the most common diseases in the western world and their prevalence in the adult population in central and northern Europe and the United states ranges from about 30% to almost 50% [1–3].

Development of allergies is associated with genetic and environmental factors [4–12].

While atopy increased markedly in the second half of the twentieth century, [13–18] the development of atopy prevalence has been controversially discussed in recent years. Whereas some studies noted a plateauing of this trend for children and adolescents, [19–21] other data documented a further slight increase of atopy prevalence [2, 12, 22, 23]. The mechanism leading to these discordances remains unclear.

In this study we hypothesised that the observed variation was due to the composition of different risk profiles for sensitization of participants that were analysed in the various studies. To examine this hypothesis, the aim of this study was to document the time course of sensitization and polysensitization in a more homogenous selected young adult population with a probable accumulation of risk factors for allergic disorders including higher socio-economic status, good education and urban life [9, 11, 12]. We analyzed the temporal trend of atopy prevalence, sensitization/polysensitization pattern and associated clinical symptoms of 1683 Swiss medical students from nine 3rd year cohorts between 2007 and 2015.

## Methods

The local Ethical Review Board of Zurich assessed the protocol of this study and offered a waiver (#8-2016). The study strictly adhered to the principles of good clinical practice and the ethical standards outlined in the Declaration of Helsinki [24]. The reporting guidelines for observational studies (STROBE) were applied [25]. All participants were verbally informed and gave their oral informed consent when in handing in the questionnaire and their blood test results.

### Participants

From 2007 to 2015, all third year medical students at the University of Zurich attended the practical course in immunology and were included, forming nine annual cohorts. No exclusion criteria were imposed. In Switzerland, medical students usually study for 6 years after completing secondary school at an age of 18 years on average. Zurich University has the largest medical school in Switzerland with about 220 students per clinical year (followed by Berne, Basel and Lausanne). About 10% of the students come from abroad. The number of inhabitants living in the catchment area for medical students at the University of Zurich is approximately 3 million. This corresponds to about 40 percent of the Swiss population. The proportion of female students has steadily increased and surpassed the 50% margin in 2009.

### Data collection

From all participants we secured information on age, gender and clinical symptoms, including rhinoconjunctivitis, asthma and eczema. Each student tested his or her blood during the practical course in immunology using the ImmunoCAP Rapid test kit, a semi-quantitative point-of-care test (Thermo Fisher Scientific Inc, Waltham MA, USA) according to the manufacturer’s instructions and a short film sequence (http://www.phadia.com/en/Products/Allergy-testing-products/ImmunoCAP-Rapid). In addition, senior staff of the University Clinic for Immunology supervised the testing. We entered anonymised personal data and the results from blood tests into an electronic database for statistical analysis.

Specific IgE antibodies against the 10 common aeroallergens cat dander (e1), birch (t3, *Betula verrucosa*), mugwort (w6, *Artemisia vulgaris*), timothy grass (g6, *Phleum pratense*), cockroach (i6, *Blatella germanica*), dog dander (e5), olive (t9, *Olea europaea*), wall pellitory (w21, *Parietaria judaica*), house dust mite (d1, *Dermatophagoides pteronyssinus*) and mould (m6, *Alternaria alternata*) were measured. The 2007 test (ImmunoCAP Rapid wheeze/rhinitis child) contained egg white (f1) and cow’s milk (f2) instead of cockroach and mould.

### Definition of sensitization and allergic diseases

We define atopy as a sensitization to any of the tested allergens, monosensitization as sensitization to only one allergen, and polysensitization as sensitization to two or more of the tested allergens. Sensitization to olive pollen was interpreted as a sensitization to ash pollen due to a cross-reaction of the sIgE antibodies [26].

We evaluated the frequency of the most common IgE-mediated allergic diseases, namely rhinoconjunctivitis, asthma and eczema [27]. The occurrence of atopic diseases was assessed by self-reported answers to the questions: “Do you suffer from seasonal/perennial rhinoconjunctivitis, asthma or eczema?”. We expected that 3rd year medical students have the required medical knowledge to answer these questions.

### Statistical analysis

Interval scaled variates were summarized with means and standard deviations or medians and interquartile ranges (IQR), where appropriate. Dichotomous variates were described as ratios and percentages. We assumed that the composition of students from rural and urban areas and the average socioeconomic status remained constant during the observation period. Under this premise, the nine cohorts were combined for further analysis and an indicator variate was created for each cohort.

The overall prevalence of atopy in each cohort and the average annual increase was assessed using a linear mixed model allowing a random intercept for each cohort. In exploratory analyses we then assessed the temporal changes of ten individual allergens.

We performed all analyses using the Stata 14.2 statistics software package (StataCorp LP, College Station, TX, USA).

## Results

From 1683 third-year medical students, the questionnaire and the ImmunoCAP Rapid test result was available in 1513 participants (89.9% of the study population). The median cohort size were 215 students, (interquartile range 193–222) and the proportion of female students ranged from 43.7–62.2%. Mean age ranged from 22.4 to 23.3 years. In ten students, data about gender was missing.

### Comparison of cohorts

Atopy was present in 604 (39.9%) participants. The most common allergen in our study population was timothy grass, with a prevalence of 27.5%, followed by house dust mite 17.5%, birch 15.3%, olive/ash 11.8% and cat dander 7%. Sensitization to mugwort, alternaria, parietaria, dog dander and cockroach were less common, with rates below 3% for each. More men than women were atopic (odds ratio (OR) 1.53 (95% CI 1.24–1.89), p < 0.001). Accordingly, males were significantly more sensitized against birch, timothy grass, olive/ash and house dust mite. Of all tested subjects, 22.8% (n = 345) were polysensitized and 17.1% (n = 259) monosensitized. Male subjects were significantly more polysensitized than females (see Tables 1, 3).

**Table 1:** Results from the ImmunoCAP rapid test: frequency of atopy and sensitization to different allergens and distribution of non-, mono- and poly-sensitized participants

	Prevalence n = 1513	Women n = 856	Men n = 647	Missing n = 10
Atopy	604 (39.9%)	305 (35.6%)	297 (45.9%)	2
Monosensitization	259 (17.1%)	134 (15.7%)	124 (19.2%)	1
Polysensitization	345 (20.5%)	171 (18.1%)	173 (26.7%)	1
Two allergens	128 (8.5%)	67 (7.8%)	61 (9.4%)	
Three allergens	111 (7.3%)	55 (6.4%)	56 (8.7%)	
≥ 4 allergens	106 (7.0%)	49 (5.7%)	56 (8.7%)	1
Cat dander (e1)	106 (7.0%)	64 (7.5%)	41 (6.3%)	1
Birch (t3)	231 (15.3%)	106 (12.4%)	124 (19.2%)	1
Mugwort (w6)	42 (2.8%)	23 (2.7%)	19 (2.9%)	
Timothy grass (g6)	416 (27.5%)	212 (24.8%)	202 (31.2%)	2
Cockroach (i6)	17 (1.1%)	8 (0.9%)	9 (1.4%)	0
Dog dander (e5)	25 (1.7%)	17 (2.0%)	8 (1.2%)	0
Olive (t9)	179 (11.8%)	86 (10.1%)	93 (14.4%)	0
Parietaria (w21)	29 (1.9%)	12 (1.4%)	17 (2.6%)	0
House dust mite (d1)	265 (17.5%)	127 (14.8%)	137 (21.2%)	1
Mold (*Alternaria*, m6)	28 (1.9%)	13 (1.5%)	15 (2.7%)	0

Gender information was missing in ten students

From the 604 atopic students, 76.6% were symptomatic of allergic-related disorders, such as rhinoconjunctivitis, asthma or eczema. The most frequent clinical symptom was seasonal rhinoconjunctivitis (68.2%), followed by asthma (20.8%), eczema (17.9%) and perennial rhinoconjunctivitis (9.4%). From the 909 non-atopic students, 16.9% reported seasonal rhinoconjunctivitis, 3.6% perennial rhinoconjunctivitis, 5.7% asthma and 12.4% eczema (Table 2).

**Table 2:** Comparison of reported symptoms among the group of students with vs. without atopy

	Subjects without atopy (n = 909)	Subjects with atopy (n = 604)	*p* value
Symptomatic	141 (15.5%)	463 (76.6%)	< 0.001
Eczema	17 (12.4%)	83 (17.9%)	0.003
Seasonal rhino-conjunctivitis	24 (16.9%)	316 (68.2%)	< 0.001
Perennial rhino-conjunctivitis	5 (3.6%)	44 (9.4%)	< 0.001
Asthma	8 (5.7%)	96 (20.8%)	< 0.001

### Time trends

**Fig. 1:**
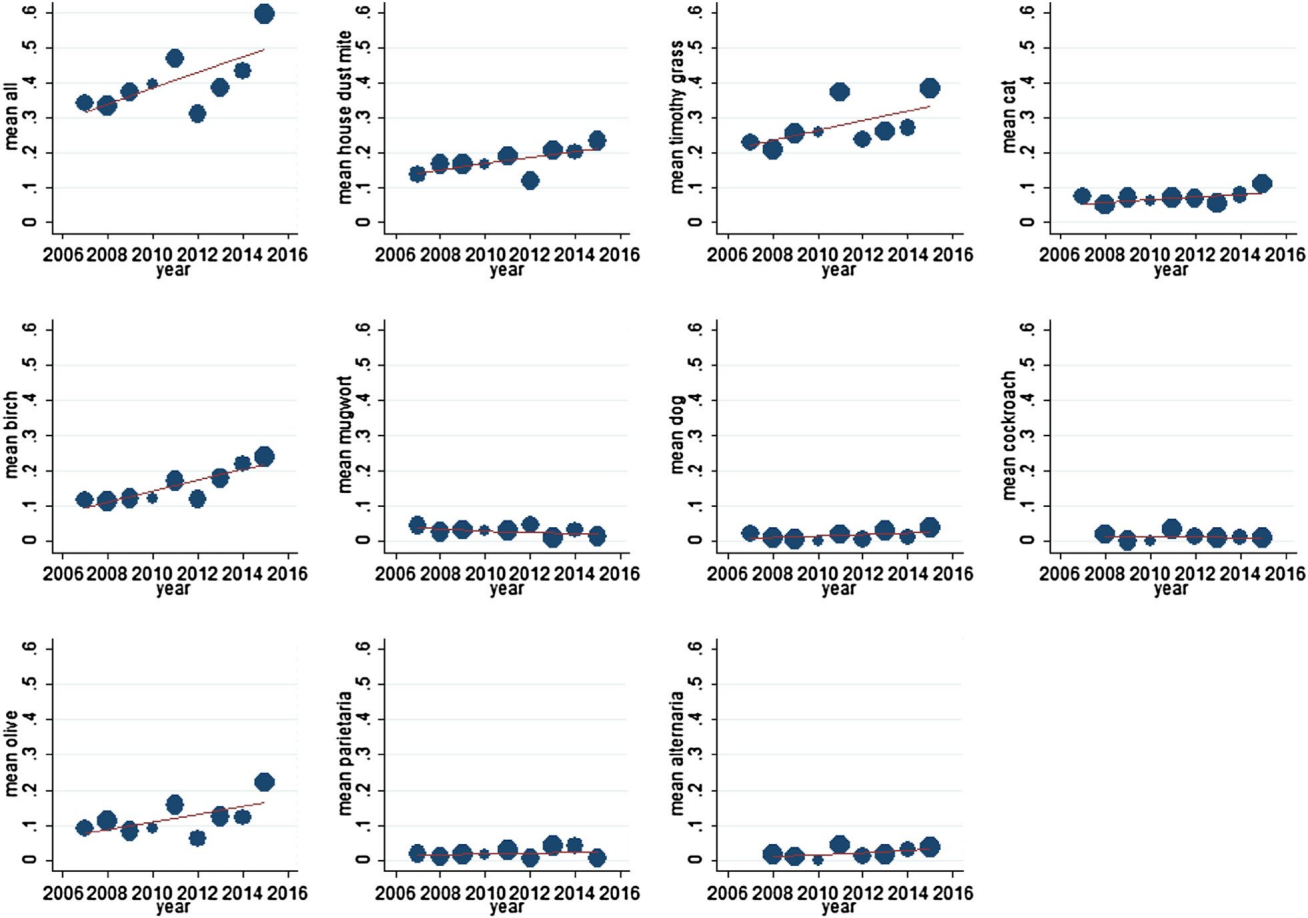
Overall (upper left) and allergen specific trend across the nine cohorts of students, in percentage of the sensitized population. House dust mites, timothy grass- and birch pollen are responsible for the overall increase

**Fig. 2:**
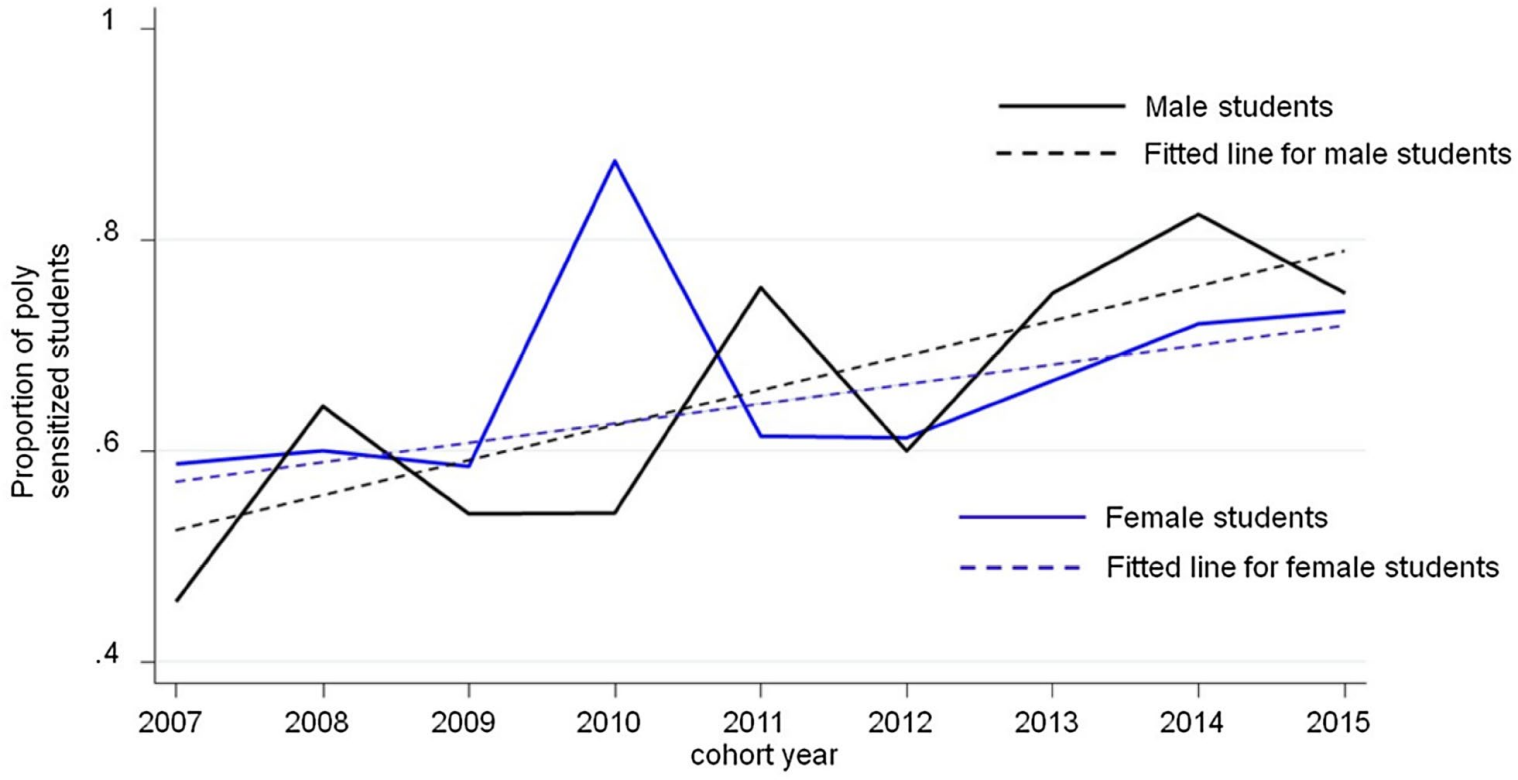
Overall and stratified trend for polysensitization among students with atopy across the nine cohorts from 2007 to 2015

Overall, we observed an average annual 2.25% increase of sensitization (95% CI 2.13–2.36%), p < 0.001. House dust mite, timothy grass- and birch-pollen were responsible for the overall increase. For details, see Fig. 1. The proportion of polysensitization slightly increased during the 9 years of observation and was more pronounced in male subjects compared to women (Table 3; Fig. 2).

**Table 3:** Proportion of sensitized students who are polysensitized per year cohort and gender

Cohort year	Polysensitized male among sensitized male (%)	Polysensitized female among sensitized female (%)
2007	14 (42.4)	17 (54.8)
2008	24 (61.5)	16 (53.3)
2009	18 (51.4)	21 (55.3)
2010	8 (42.1)	6 (85.7)
2011	32 (72.7)	33 (60.0)
2012	14 (46.7)	13 (52.0)
2013	21 (67.7)	28 (59.6)
2014	15 (68.2)	9 (42.9)
2015	27 (58.7)	28 (54.9)

## Discussion

In this selected population of Swiss, 3rd year medical students, we were able to document a slight increase of sensitization and polysensitization for common aeroallergens during the 9 years of observation. Timothy grass pollen, house dust mites and tree pollen are the most prevalent allergens and they were the main drivers for the overall increase of sensitization in the described study-population. Compared to cohorts in northern Europe, prevalence of cat dander sensitivity is relatively low. The extent of sensitization in our cohort corresponds to the results found in Germany, the USA, and previous Swiss studies [2, 3, 23, 28, 29]. This is most probably due to climatic distinctions and exposure frequency.

While atopy increased markedly in the second half of the twentieth century, [13, 16–18] data about atopy prevalence have been more controversial in recent years. Some studies indicate a flattening and stabilization of atopy prevalence in Switzerland and other westernized countries [19–21, 30] whereas other data noted a further increase [12, 23, 31]. Our study describes a selected population of highly educated young adults, living predominantly in urban areas with a high social status. All these factors are described to have the potential to increase the risk of developing atopy and allergic diseases [9, 11, 12, 23, 32]. This exposure to several risk factors might explain the slight increase of atopy prevalence in our cohorts over 9 years.

The atopy prevalence of 39.9% and the gender distribution with male subjects suffering more often from atopy and being more often polysensitized than women, is in line with other studies from industrialized countries in central-, and northern Europe, and the United States [2, 3, 28, 29, 33–35]. As documented in other studies, polysensitization is more prevalent than monosensitization [36–38] and more pronounced in men (compare Tables 1, 3). Polysensitization is associated with clinical manifestation of allergic diseases, especially with asthma [38, 39]. Therefore, our findings with an increase of sensitization and polysensitization, in parallel, support an overall increase of symptomatic atopic patients in the described cohorts.

Rhinoconjunctivitis, asthma and eczema are a complex of disorders, which often coexist and therefore seem to have a causal relationship. Normally associated with atopy, this symptom complex also occurs in non-atopic subjects [40] as shown in Table 2.

### Strength and limitations

In this study it was possible to compare cohorts with an accumulation of similar risk factors for the development of allergic sensitization and related disorders over several years. The lack of detailed information about the subjects’ genetic background, place of birth, and the time point when sensitization and/or allergic symptoms were first diagnosed is a downside of this study. Therefore, we were unable to explore the extent to which these parameters modified the effects reported here. Arguably, spurious differences in the composition of these parameters across cohorts, which we were unable to correct in the analysis, introduced bias. Nevertheless, due to the lack of an obvious mechanism leading to substantial changes in the composition of cohorts over time, we consider the results valid. Self-reporting of symptoms carry the risk of reporting bias. We cannot fully rule-out that such bias occurred in our study. On the other hand, it can be expected that 3rd year medical students have the necessary medical knowledge to indicate whether they are suffering from seasonal/perennial rhinoconjunctivitis, asthma or eczema.

## Conclusions

Our findings suggest that the trend for an increase in atopy prevalence and polysensitization is still on the rise in young adults with certain risk factors. Further investigations in selected cohorts with defined risk factors for atopic diseases could help to elucidate the mechanisms of sensitization. Allergic diseases rhinoconjunctivitis, asthma and eczema are associated with atopy but also coexist in non-atopic subjects.
